# Redesigning the regulatory pathway to enhance cellulase production in *Penicillium oxalicum*

**DOI:** 10.1186/s13068-015-0253-8

**Published:** 2015-04-23

**Authors:** Guangshan Yao, Zhonghai Li, Liwei Gao, Ruimei Wu, Qinbiao Kan, Guodong Liu, Yinbo Qu

**Affiliations:** State Key Laboratory of Microbial Technology, Shandong University, Jinan City, Shandong Province 250100 China; National Glycoengineering Research Center, Shandong University, Jinan City, Shandong Province 250100 China; Qingdao Vland Biotech Group Co. Ltd., Shandong Expressway Mansion, Miaoling Road, Qingdao, Shandong China

**Keywords:** *Penicillium oxalicum*, Cellulase, Transcription factor, Beta-glucosidase, Genetic engineering, Secretome

## Abstract

**Background:**

In cellulolytic fungi, induction and repression mechanisms synchronously regulate the synthesis of cellulolytic enzymes for accurate responses to carbon sources in the environment. Many proteins, particularly transcription regulatory factors involved in these processes, were identified and genetically engineered in *Penicillium oxalicum* and other cellulolytic fungi. Despite such great efforts, its effect of modifying a single target to improve the production of cellulase is highly limited.

**Results:**

In this study, we developed a systematic strategy for the genetic engineering of *P. oxalicum* to enhance cellulase yields, by enhancing induction (by blocking intracellular inducer hydrolysis and increasing the activator level) and relieving the repression. We obtained a trigenic recombinant strain named ‘RE-10’ by deleting *bgl2* and *creA*, along with over-expressing the gene *clrB*. The cellulolytic ability of RE-10 was significantly improved; the filter paper activity and extracellular protein concentration increased by up to over 20- and 10-fold, respectively, higher than those of the wild-type (WT) strain 114-2 both on pure cellulose and complex wheat bran media. Most strikingly, the cellulolytic ability of RE-10 was comparable with that of the industrial *P. oxalicum* strain JU-A10-T obtained by random mutagenesis. Comparative proteomics analysis provided further insights into the differential secretomes between RE-10 and WT strains. In particular, the enzymes and accessory proteins involved in lignocellulose degradation were elevated specifically and dramatically in the recombinant, thereby confirming the importance of them in biomass deconstruction and implying a possible co-regulatory mechanism.

**Conclusions:**

We established a novel route to substantially improve cellulolytic enzyme production up to the industrial level in *P. oxalicum* by combinational manipulation of three key genes to amplify the induction along with derepression, representing a milestone in strain engineering of filamentous fungi. Given the conservation in the mode of cellulose expression regulation among filamentous fungi, this strategy could be compatible with other cellulase-producing fungi.

**Electronic supplementary material:**

The online version of this article (doi:10.1186/s13068-015-0253-8) contains supplementary material, which is available to authorized users.

## Background

Plant biomass-based fuels and chemicals offer an appealing and long-term solution as a replacement to fossil fuels [1]. Enzymatic hydrolysis of biomass to fermentable sugar is a key step for biofuel refinery. However, high-cost cellulases are the major bottlenecks to economically competitive cellulose-to-biofuel conversion [2]. *Trichoderma reesei* has always been used as the workhorse to produce cellulase cocktails, and multiple strategies have been applied to improve its enzyme yields to lower production costs [3,4]. Fungi from *Penicillium* genus have recently attracted a great deal of research attention, and they are considered as potential alternatives to *T. reesei* for second-generation biofuel production [5].

*Penicillium oxalicum* (previously named as *Penicillium decumbens*) was selected for its strong cellulolytic ability in saprophytic condition. It has been under investigation for more than 30 years in China [6]. Whole genome sequencing revealed that the fungus obtains a unique lignocellulose-degrading enzyme arsenal during evolution. Data from comparative genomics analysis demonstrates that this fungus has higher number and more types of genes encoding hemicellulases, as well as genes encoding cellulose binding domain (CBM) containing proteins, than other cellulolytic fungi, such as *T. reesei* and *Aspergillus niger* [6]. *P. oxalicum* also exhibits higher beta-glucosidase activity in its enzyme system than that of *T. reesei*, which is shared by many other *Penicillium* species [7]. Random mutagenesis and process engineering had been successfully applied to *P. oxalicum* for improving cellulase production [8-10]. One of these mutants, JU-A10-T, with a maximum volume productivity of 160 U L^−1^ h^−1^, was developed and utilized in industrial scale cellulase production since 1996 in China [10]. Similar to *T. reesei*, breakthroughs are required for maximizing the yields and minimizing the cost to make the biomass-based fuel a powerful competitor to fossil fuel [4].

In recent years, studies based on omics and system biology have provided us with crucial information to understand the biology of lignocellulose-degrading enzyme production in cellulolytic fungi [10,11]. It is widely accepted that the expressions of almost all genes encoding lignocellulose-degrading enzymes are triggered by inducer released from complex plant polysaccharides and regulated by transcription factors in a coordinated manner [12,13]. Systematic investigation of the regulatory network for cellulase gene expression has led to the identification of many transcription factors. Some of these transcription factors are found in all cellulolytic fungi, but others are genus- or species-specific. Clr2/ClrB protein, containing Zn2Cys6-type DNA binding domain, functions as a positive regulator for cellulase gene expression in ascomycete fungi, and its deletion incurs defects in growth and cellulase activity to nearly undetectable level when cultured on Avicel medium in *Neurospora crassa* and *Aspergillus nidulans* [14]. Similarly, a homolog of *clr-2/clrB* in *P. oxalicum* was found to be necessary for efficient cellulase production, and its over-expression resulted in a significant increase in cellulases at both transcriptional and protein levels (unpublished data).

In addition to the transcriptional activation mechanism, carbon catabolite repression (CCR) triggered by glucose and other easily metabolized carbon sources exists widely in *Saccharomyces cerevisiae* and filamentous fungi [15]. In cellulolytic fungi, the CCR mechanism is mediated mainly by the transcription factor CreA/Cre1, which suppresses the expression of a majority of cellulase and hemicellulase genes in *A. niger*, *T. reesei*, *N. crassa*, and *P. oxalicum* [9,12,16,17].

Recently, investigations on the induction mechanism uncover the potential targets in the upstream of transcriptional regulators. Cellobiose, a hydrolysate from plant cell wall materials, is assumed to be the natural inducer for cellulase gene expression in *N. crassa* and *T. reesei* when growing on cellulose medium [18,19]. The level of intracellular cellobiose is balanced by the import by cellobiose transporter and intracellular beta-glucosidase hydrolysis. In *P. oxalicum*, a previous study demonstrated that the deletion of gene *bgl2* encoding the major intracellular beta-glucosidase results in significantly improved cellulase production [20].

Genetically modifying its regulatory factor rather than the target gene is an efficient and promising strategy in the improvement of complex cellulase mixture in filamentous fungi. It is reported that the deletion or replacement of gene *creA* with truncated mutant variant enhances the cellulolytic enzyme production capacity in *T. reesei* [21]. Point mutation of the activator *xyr1* in *T. reesei* or mis-expression of the *clr-2* in *N. crassa*, respectively, exhibits inducer-independent production of cellulolytic enzymes, but not in *A. nidulans* [22,23]. Taken together, although some of these aforementioned regulatory factors have been genetically engineered in cellulolytic fungi, their effects, however, in improving the productivity of cellulolytic enzymes are highly limited.

In the present study, we developed a systematic strategy to redesign the regulatory network (RE) to enhance cellulase production by combinatorial manipulation of three important regulators in *P. oxalicum*: over-expressed *clrB* for enhancing induction, deleted *bgl2* for inducer accumulation, and deleted *creA* for derepression. As a result, the cellulolytic ability of the triple-gene recombinant RE-10 was significantly improved. Comparative analysis of the secretomes between RE-10 and wild type (WT) provided more insights into the cellulase system of *P. oxalicum* and alterations caused by the strain rational engineering.

## Results

### Designing a systematic strategy to genetically modify the cellulolytic fungus *P. oxalicum* for improving cellulolytic enzyme expression

**Figure 1:**
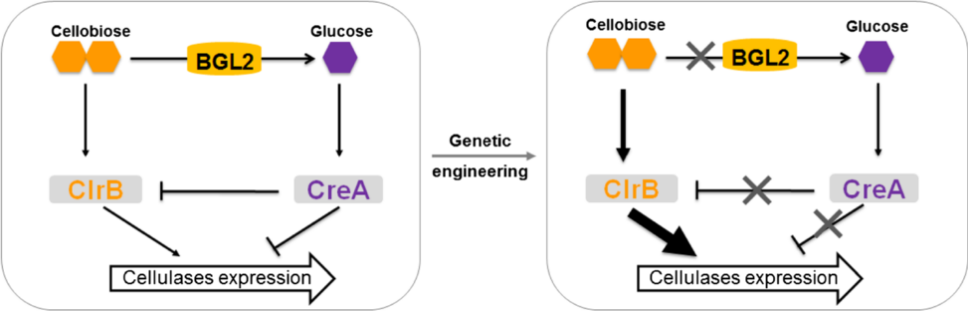
Scheme of a systematic strategy for the genetic modification of *P. oxalicum.* In the WT strain, the regulatory network for cellulase expression is balanced between the induction from cellobiose, which is the substrate of BGL2 and mediated by ClrB, and repression from glucose, which is the product of BGL2 and mediated by CreA (left). CreA (deletion), ClrB (over-expression), and BGL2 (deletion) were genetically engineered for the amplification of the induction and the elimination of CCR for maximizing the output of cellulase expression (right).

To improve the production of the lignocellulolytic enzymes, a systematic approach was developed for genetically modifying the cellulolytic fungus *P. oxalicum* (Figure 1). Clr-2, its ortholog in *P. oxalicum* is ClrB, is essential for inducing cellulase expression and conserved in ascomycete fungi [14]. Therefore, as the first target, the level of ClrB was increased by constitutively over-expressing the gene with the promoter *gpdA* from *A. nidulans* [24]. Multiple transformants with *clrB* expression cassette insertion and resistant to pyrithiamine were screened, and one of them was verified by PCR and Southern blot (Additional file 1: Figure S1A). As expected, the transcript level of gene *clrB* increased by up to 100 and 12 times at cellulose induction 4 and 22 h, respectively, compared with those of WT (Additional file 2: Figure S2A).

Previous work in our laboratory demonstrated that the major intracellular beta-glucosidase BGL2 plays a negative role in the induction of cellulases and xylanase, thereby leading to a hypothesis that the deletion of *bgl2* facilitates the accumulation of intracellular cellobiose [20], which is the natural stimuli for inducing the expression of cellulolytic enzyme genes. As a consequence, a knockout cassette was constructed to replace the gene *bgl2* with the marker gene *hph* in the above mutant with *clrB* over-expression. More than ten transformants resistant to hygromycin B were obtained and verified by PCR (data not shown). One of those transformants, confirmed by Southern blot (Additional file 1: Figure S1B), was selected for further genetic engineering. No transcription of *bgl2* was further confirmed by real-time quantitative polymerase chain reaction (qRT-PCR) (Additional file 2: Figure S2B).

In addition to the induction mechanism, the expressions of cellulase and hemicellulase genes are repressed when the preferred carbon sources are available, which is mediated by transcription factor CreA. To overcome repression, we deleted the gene *creA* by using the gene bar as the marker. Three transformants with resistance to herbicide bialaphos on a selectable plate were selected [25] and then verified by PCR. One of three transformants was confirmed by Southern blot (Additional file 1: Figure S1C) and named RE-10 in the following research. The lack of transcription of the gene *creA* was further confirmed by qRT-PCR (Additional file 2: Figure S2C).

In summary, the regulatory pathway was modified to reconstruct the regulatory network of expression of cellulolytic enzyme genes, by simultaneously strengthening induction and relieving repression. Three key factors involved in this network were genetically modified (Figure 1).

### Regulatory pathway redesigning substantially enhanced cellulolytic enzyme production in the mutant RE-10

**Figure 2:**
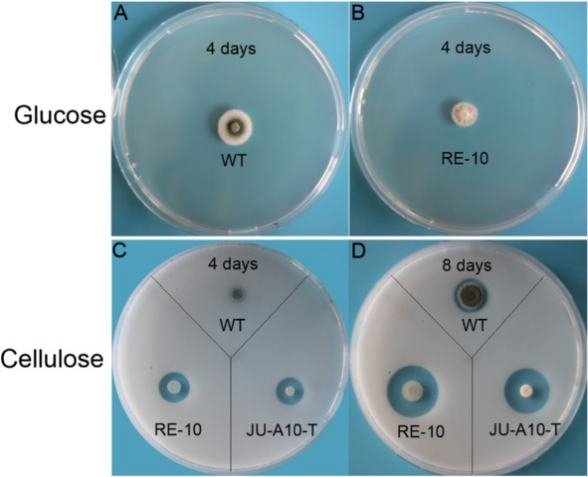
Cellulolytic phenotypic analysis of the recombinant RE-10. Equally harvested conidia of WT and RE-10 or JU-A10-T were inoculated on both glucose medium (**A** (WT), **B** (RE-10)) or cellulose medium **(C and D)** for 4 and 8 days, respectively, and then photographed.

Comparative genomics analysis between *P. oxalicum* WT strain 114-2 and cellulolytic enzyme hyper-producer JU-A10-T demonstrates that functional mutation occurred in the transcription factor CreA, and the promoter of *cbh1* contributes greatly to the high-producing phenotype [9]. Three key factors which control the expression of genes encoding lignocellulose-degrading enzymes were deleted or over-expressed in this study to investigate whether these manipulations could improve the cellulase production as classical mutagenesis. To determine the effect of the genetic modifications on *P. oxalicum*, phenotypic and cellulolytic ability analyses were conducted. Equivalent fresh spores of the WT, RE-10, and JU-A10-T strains were inoculated on plates with 2% glucose or 2% cellulose as the sole carbon source for 4 or 8 days, respectively. As shown in Figures 2A and B, slightly smaller colonies and less conidia were observed in RE-10 than those in WT strain when grown on glucose medium. However, the morphological defects became less evident when cultured on cellulose medium. These morphological changes coincided with our previous observations of the repressor gene *creA* deletion in *P. oxalicum* WT strain 114-2 [9]. Similar morphological alterations caused by *cre1*/*creA* mutant were widely reported in other filamentous fungi. For example, deletion of the gene *cre-1* in *N. crassa* led to grow slower and denser than the parental strain under glucose, sucrose, or xylose conditions [16]. Moreover, the *creA* mutant displayed reduced growth, conidia under repressing conditions (including glucose, ethanol, and galactose) in *A. nidulans* [26], as well as in *Acremonium cellulolyticus* [27].

As expected, a considerably great and clear cellulolytic halo was observed around the colonies of RE-10 but not in WT after 4 days of incubation, and this halo was much more pronounced after 8-day growth (Figures 2C and D). Beyond we expected, the diameter of the cellulolytic halo of RE-10 was much greater than that of the industrial strain JU-A10-T (1.1 ± 0.1 and 1.9 ± 0.1 cm at days 4 and 8 for RE-10 compared with 0.9 ± 0.1 and 1.6 ± 0.1 cm at days 4 and 8 for JU-A10-T) (Figures 2C and D).

**Figure 3:**
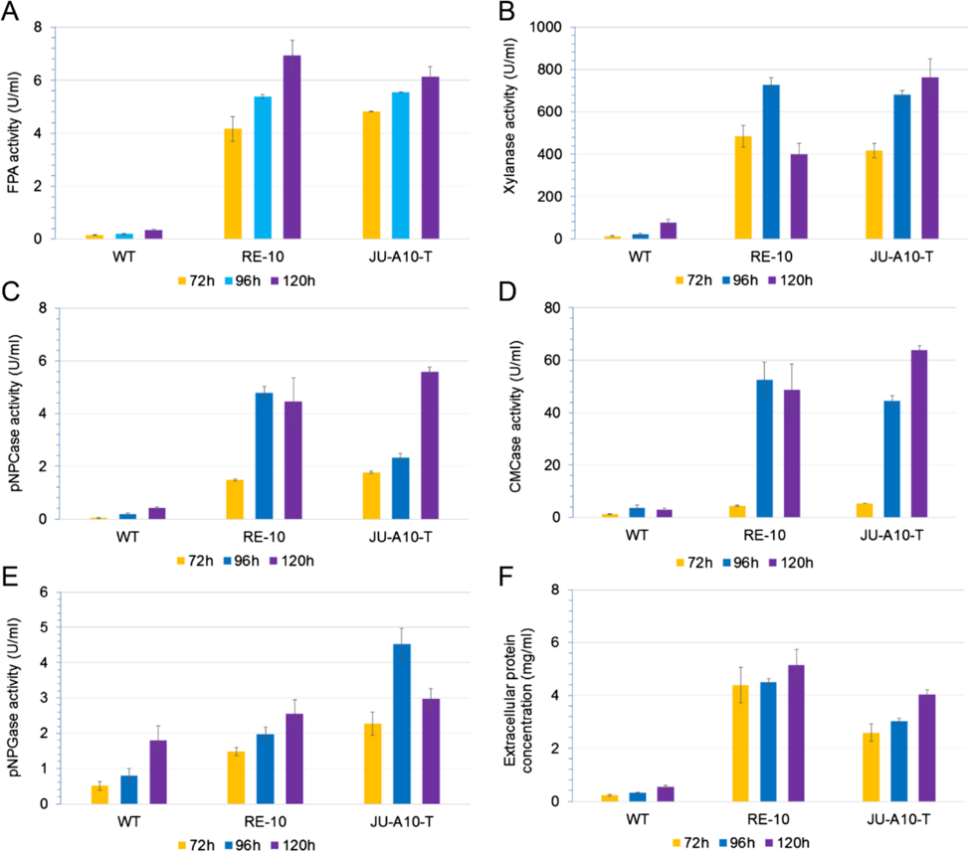
Comparative cellulolytic activity assay in wheat bran medium. The FPA **(A)**, xylanase **(B)**, *p*NPCase **(C)**, CMCase **(D)**, *p*NPGase activities **(E)**, and protein **(F)** of WT, RE-10, and JU-A10-T on wheat bran medium were determined at 72, 96, and 120 h. The values show the mean of three biological replicates, and the error bar indicates the standard deviation.

On the other hand, the cellulolytic enzyme activities were evaluated comprehensively for RE-10 and WT when cultured on cellulose medium, along with JU-A10-T when cultured on wheat bran medium. The later medium is a cheap and ideal mixture optimized for cellulase production by *P. oxalicum*. The levels of filter paper activity (FPA, representing overall cellulase activity), CMCases (endoglucanase), *p*NPCases (cellobiohydrolase), *p*NPGases (beta-glucosidase), xylanase activities, and total extracellular protein concentration in the culture supernatant of all strains, including WT, RE-10, and JU-A10-T were determined. The results (as shown in Additional file 3: Figure S3) showed that almost all the enzymes assayed were elevated substantially in RE-10 compared with those in WT on cellulose media, which were consistent with the phenotypic analyses above, except beta-glucosidases. As for the comparison between WT and RE-10, the levels of FPA, *p*NPCase, CMCase, xylanase activities, and extracellular total protein were approximately 20-, 50-, 50-, 16-, and tenfold higher, respectively, in RE-10 than those in the wild-type strain (Additional file 3: Figures S3A-E). Notably, a remarkable decrease in FPA was observed in RE-10 at 108 h than that at 96 h, which may be due to product feedback inhibition caused by reduction of *p*NPGase activity in the late stage of fermentation (Additional file 3: Figures S3A and S3D). This founding was in accordance with a common consensus that beta-glucosidases play an important role in eliminating product inhibition of cellobiohydrolases and endoglucanases [28,29]. However, the similar results were not detected in wheat bran medium (Figure 3A).

Complex carbon sources from plant materials are more efficient than pure cellulose in induction of expression of lignocellulose-degrading enzymes. Expectedly, when cultured on wheat bran medium, much higher cellulase activities were detected for both WT and RE-10. In particular, FPA reached 6.94 ± 0.57 U/mL in the RE-10 strain, which was 27-fold higher than that of WT (*P* value <0.01). Moreover, *p*NPCase, CMCase, xylanase activities, and extracellular protein level increased by 10-, 16-, 5-, and 10-fold in RE-10 compared with those of WT, respectively (Figure 3).

**Figure 4:**
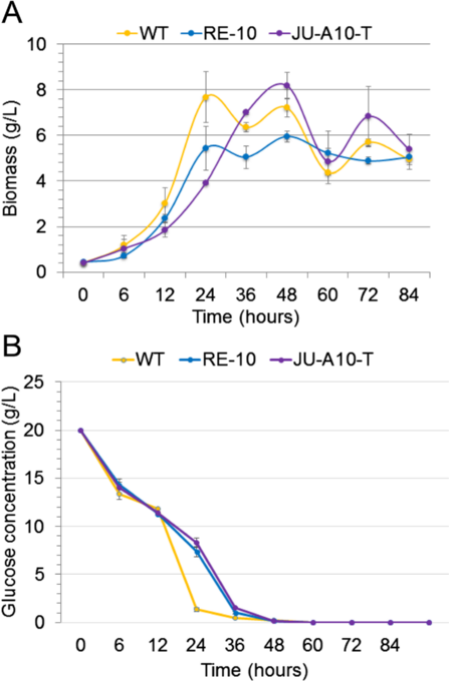
Growth kinetics and glucose utilization. **(A)** Growth kinetics of WT (orange), RE-10 (blue), JU-A10-T (purple) in 2% glucose medium; **(B)** The concentration of residual glucose of WT (orange), RE-10 (blue), JU-A10-T (purple) at the sampling points.

Inspiringly, our results demonstrated that the cellulolytic activities, including FPA, xylanase, CMCase, *p*NPCase, and *p*NPGase, of the recombinant strain RE-10 were comparable to those of the industrial strain JU-A10-T (Figures 3A to E), which were coherent with the above cellulolytic halo assay. It is generally viewed that the production of hydrolytic enzymes is closely associated with the fungal biomass. Thereby, we questioned that whether the improvement in cellulase synthesis in RE-10 was due to its higher biomass level. Then, we examined both the growth kinetics and glucose utilization of WT, RE-10, and JU-A10-T strains, and the results were showed in Figure 4. We observed that JU-A10-T accumulated the slightly higher level of biomass (approximately 8 g/L), relative to the WT (7.5 g/L) in the end (Figure 4A), although at slightly low growth and glucose utilization rates (Figure 4B). Clearly, RE-10 (about 6 g/L) had a lower biomass formation (*P* <0.05) than that of WT or JU-A10-T (Figure 4A), although consumed the glucose at the same rate as the WT (Figure 4B). Collectively, our results confirmed that higher cellulolytic activities in RE-10 did not correlate with the biomass level.

**Figure 5:**
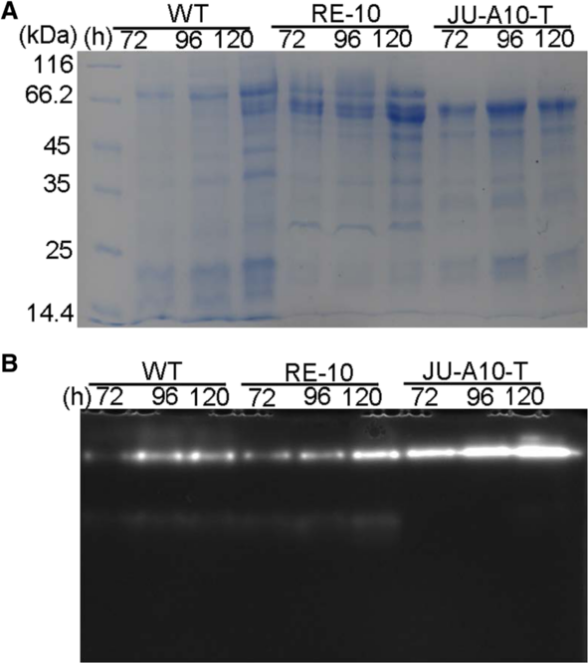
SDS-PAGE and zymography analysis of the secreted protein. **(A)** SDS-PAGE analysis of the culture supernatants of the WT, RE-10, and JU-A10-T. **(B)** Activity staining was used to measure the CBH activity of the WT (left), RE-10 (middle), and JU-A10-T (right).

Subsequently, the supernatants from the WT, RE-10, and JU-A10-T strains cultured in wheat bran medium were profiled by SDS-PAGE. When equal protein loading, the protein profile secreted by RE-10 resembled that of WT, differed from that of JU-A10-T (Figure 5A). Significantly, much more protein bands were detected in RE-10 compared with WT, especially at the range of 45 to 116 kDa, which showed good correlation with the extracellular protein concentration measurement (Figure 3F, Additional file 4: Figure S4). More importantly, dynamic zymography analysis by using 4-methylumbeliferyl-β-d-cellobioside [30] as the substrate revealed that RE-10 exhibited a cellobiohydrolase activity pattern similar to WT, and the cellobiohydrolase activity (especially band 2) significantly enhanced in 120 h relative to WT (Figure 5B). However, a significant difference could be seen in the cellobiohydrolase zymography between WT/RE-10 (two bright bands) and JU-A10-T (only one bright band).

### Substantial up-regulation in transcript levels of major cellulolytic enzyme genes analyzed by qRT-PCR

**Figure 6:**
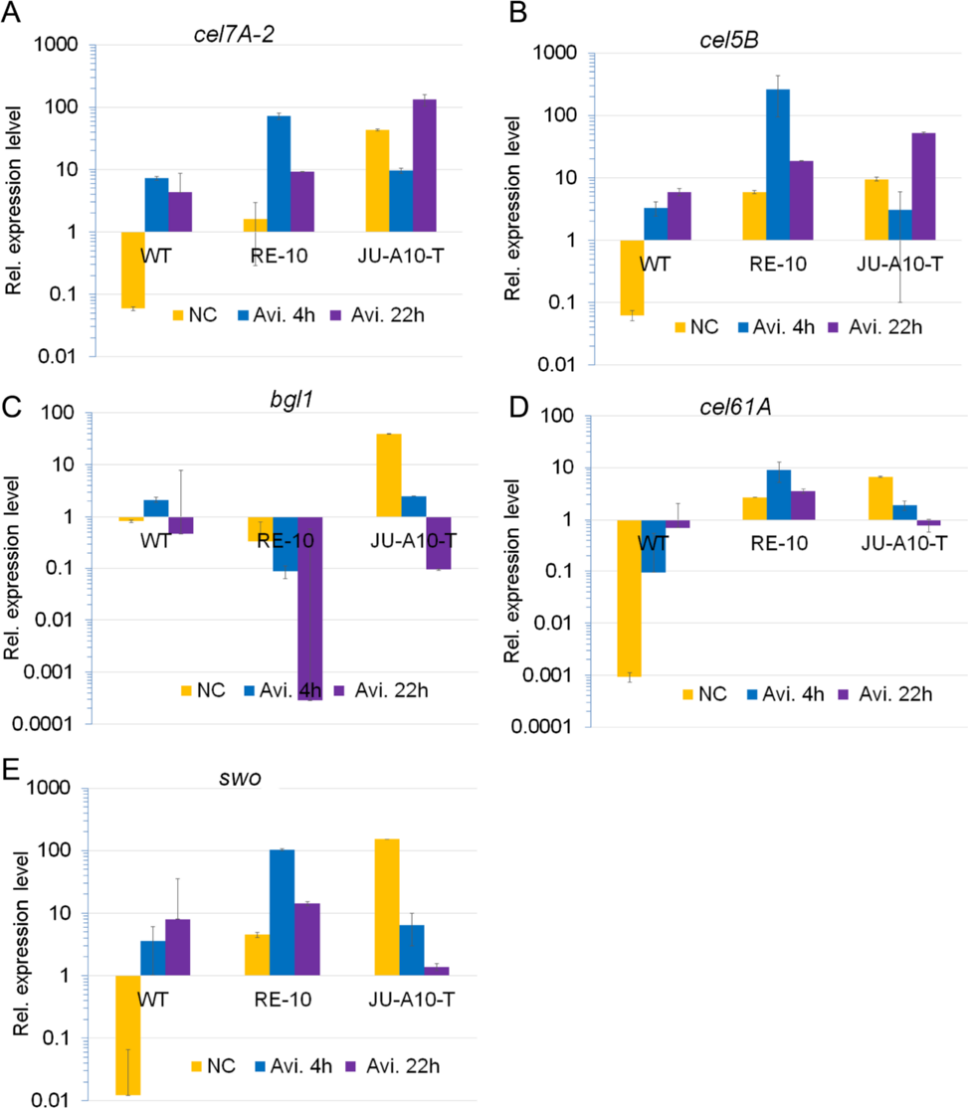
qRT-PCR analysis of the transcripts. Transcript levels of genes encoding cellulases and accessory protein including *cel7A-2* **(A)**, *cel5B* **(B)**, *bgl1* **(C)**, *cel61A* **(D)**, *swo* **(E)** in the WT (orange), RE-10 (blue), and JU-A10-T (purple) were analyzed. The values show the mean of three replicates, and the error bar indicates the standard deviation.

Compared with the WT strain 114-2, a significant improvement in cellulase yields was observed in RE-10. We wondered whether this improvement was the result from the up-regulation in transcription level. Therefore, the transcripts of major cellulases Cel7A-2, Cel5B, BGL1, Cel61A, and accessory protein swollenin are selected for qRT-PCR analyses. *P. oxalicum* 114-2, RE-10, and JU-A10-T mutant strains were pre-cultured on glucose medium for 20 h, starved for 2 h under no carbon source conditions, and transferred to cellulose medium for further induction for 4 and 22 h, respectively. The results indicated that the transcript abundance of genes encoding major cellulolytic enzymes and synergetic protein except *bgl1* (PDE_02736) in RE-10 was up-regulated significantly than that of WT under both starvation and induction conditions (Figure 6A to E), which was consistent with enzyme activity analysis above. The transcript level of *cel7A-2* (PDE_07945) increased by 27- and tenfold (*P* value <0.01) under starvation condition and 4 h of induction, respectively (Figure 6A). Meanwhile, the transcription expression level of major endoglucanase gene *cel5B* (PDE_09226) increased by over 80-fold (*P* value <0.001) both on no carbon source and 4 h of induction (Figure 6B). Especially, the transcript abundance of *cel61A* (PDE_05633) was up-regulated up to 2929-fold in RE-10 compared with that of WT strain under starvation conditions. Moreover, the transcript level of gene *PDE_02102*, which encodes swollenin destroying cellulose and enhances lignocellulose hydrolysis when supplemented to the enzyme system [31,32], was also increased by over 100-fold under starvation conditions (Figure 6E). However, the transcription expression level of the major extracellular beta-glucosidase encoding gene *bgl1* decreased significantly in RE-10 under both starvation and induction conditions (Figure 6C), which was consistent with the aforementioned enzyme activity assay, and previous reports that extracellular beta-glucosidases were regulated by a different regulatory pathway, at least in part, from other induced cellulases. Moreover, JU-A10-T substantially up-regulated the expression levels of most cellulolytic genes in both starvation condition (fold change >100, *P* value <0.01) and post-22 h induction, but not in post-4 h induction. Although it exhibited an expression pattern, which is distinct from that of RE-10 (Figure 6A to E), both RE-10 and JU-A10-T enhanced transcription expression level of most of cellulolytic genes at the same order of magnitude relative to WT.

### Comparative secretome analysis

**Figure 7:**
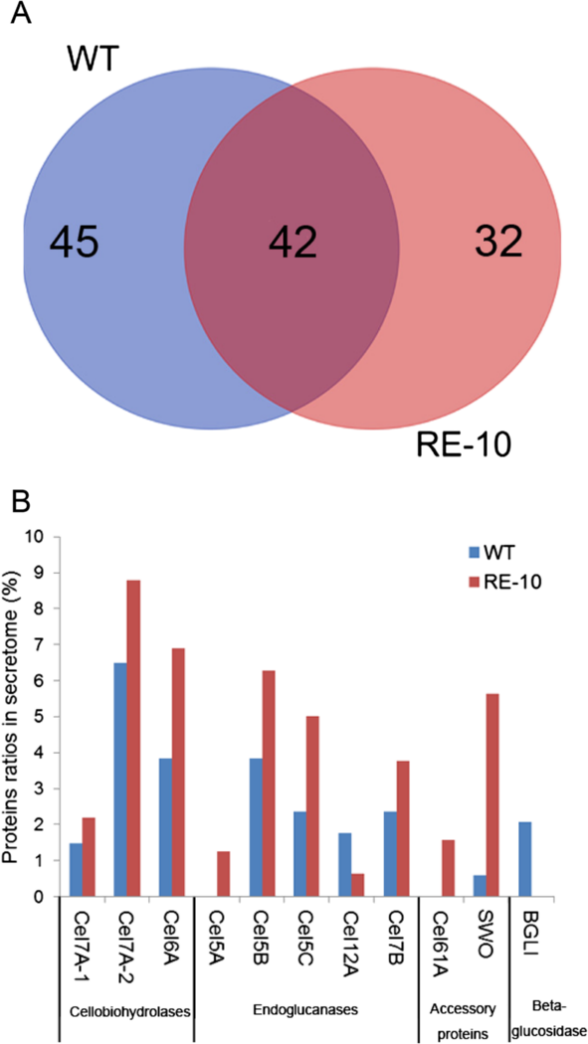
Comparative proteomics analysis between WT and RE-10. Venn diagram shows the shared secretome (overlap) and specific secretome for WT (blue) and RE-10 (red) **(A)**. The result of comparative analysis of the ratios of major cellulases and accessory proteins between WT and RE-10 is shown **(B)**.

The cellulolytic ability of the triple-gene recombinant RE-10 was significantly enhanced at both the transcript and enzyme activity levels. We assumed that a great change may occur in the secretome for RE-10 compared with that of WT strain. To explore the different profile of total secreted proteins between *P. oxalicum* 114*-*2 and RE-10 on cellulose medium, we performed high-resolution proteomics to dissect their secretomes. The supernatants of RE-10 and WT strain cultures were collected at hour 96 and analyzed by liquid chromatography-tandem mass spectrometry (LC-MS/MS). We identified a total of 157 proteins in the secretome of WT with confidence while 144 proteins were detected in that of RE-10 (Additional file 5: Table S1). Among the proteins tested, 79 of 157 had the predicted secretion signals for WT strain, while 67 of 144 were predicted to be secreted for RE-10. However, we could not rule out the possibility that the proteins without predicted secretion signals were extracellularly located; like that, 18% of secretome without classic secretion signals are detected in the secretome of *A. niger* [33]. Among those proteins with predicted secretion signals, 39 proteins were shared by both RE-10 and WT, 28 proteins were unique to RE-10, and 40 proteins were specific to WT (Figure 7A). The majority of the secretomes shared by both RE-10 and WT were cellulolytic enzymes and accessory proteins (Table 1). Core cellulases were included in the secretome induced by cellulose, which is almost consistent with the result released recently in *P. oxalicum* GZ-2 under the cellulose medium [34]. It included all the three predicted cellobiohydrolases, Cel7A-1 (PDE_5445), CBH7A-2 (PDE_07945), Cel6A (PDE_07124), and four of 11 predicted endoglucanases, Cel7B (PDE_07929), Cel5B (PDE_09226), Cel5C (PDE_09969) and Cel12A (PDE_06439). Unsurprisingly, seven hemicellulases, Xyn10A (PDE_08094), Xyn11A (PDE_02101), Xyn11B (PDE_02682), Axe1A (PDE_09278), Axe5A (PDE_04182), Man5A (PDE_06023), and Aga27A (PDE_02514), were also detected in the overlapped secretomes. It also contained one pectinase, Pga48A (PDE_04162), an amylase (PDE_01354), a swollenin (PDE_02102), a putative rhamnogalacturonan alpha-L-rhamnopyranohydrolase (PDE_09285), a putative polygalacturonase (PDE_07938), lysophospholipase (PDE_05537), a cell wall integrity and stress response component (PDE_01796), and 17 other proteins with uncharacteristic functions (Table 1). Subsequently, functional annotation of the WT- or RE-10-specific secretomes showed that a large amount of them were proteins with unidentified functions. It is worth noting that the protein PDE_00507 (an ortholog of EGII from *T. reesei*, which belongs to the GH5 family) was exclusive to RE-10, which showed relatively higher activity on cellulose than other endoglucanases from *P. oxalicum* [35]. Furthermore, a protein encoding superoxide dismutase (PDE_09399) was detected in the secretome of RE-10. Likewise, this protein was also detected in cellulase preparations from *T. reesei* (unpublished data), but its function in cellulose degradation has not yet been reported. Especially, two GH61 proteins (PDE_05633 and PDE_01261), which recently re-classified into auxiliary activities family 9 (AA9), were detected uniquely in RE-10.

**Table 1:** **Secreted proteins shared by WT and RE-10**

**Gene ID**	**Function description**	**Signal peptides**	**Coverage (WT/RE-10)**	**Total peptides (WT/RE-10)**	**Unique peptides (WT/RE-10)**	**MW [kDa]**	**Calc. pI**
PDE_01261	Putative cellulose monooxygenase	Y	4.79/24.79	1/3	1/1	36.2	5.9
PDE_01335	Hypothetical protein	Y	26.16/22.09	3/2	3/2	18.4	4.97
PDE_01354	Starch and chitin binding domain-containing	Y	6.7/23.82	6/8	6/7	42.8	6.38
PDE_01796	Hypothetical protein	Y	36.27/36.27	4/6	4/6	19.9	4.92
PDE_02101	Putative endo-beta-1,4-xylanase	Y	10.65/10.65	3/3	3/3	31.3	6.96
PDE_02102	Putative swollenin	Y	8.62/45.29	2/18	1/16	51.9	5.05
PDE_02392	Hypothetical protein	N	10.26/6.62	3/2	1/1	64.4	7.56
PDE_02514	Putative alpha-galactosidase	Y	11.64/14.69	3/3	1/2	55	6.24
PDE_02536	Hypothetical protein	Y	24.87/24.87	7/5	7/4	18.6	5.01
PDE_02682	Putative endo-beta-1,4-xylanase	Y	3.46/3.46	1/1	1/1	29.5	6.28
PDE_03112	Putative exo-beta-1,3-glucanase	Y	18.62/7.29	7/3	7/3	50.3	4.79
PDE_03255	Hypothetical protein	Y	82.31/40.82	7/3	7/3	15	5.26
PDE_03292	Chitin binding domain-containing protein	Y	4.43/4.43	1/1	1/1	27.6	4.41
PDE_03437	Hypothetical protein	Y	9.33/6.22	3/2	2/2	23.4	5.45
PDE_03452	Putative beta-1,3-glucanosyltransglycosylase	Y	2.43/4.85	1/2	1/1	57	5.22
PDE_03466	Putative rhamnogalacturonase	Y	17.01/27.13	5/7	3/5	47	6.6
PDE_03659	Hypothetical protein	N	12.17/4.52	4/2	1/1	86.7	6.61
PDE_03916	Hypothetical protein	Y	15.17/20.3	3/4	1/1	51.2	5.64
PDE_03934	Hypothetical protein	Y	19.73/18.37	3/1	2/1	15.7	6.95
PDE_04162	Putative endopolygalacturonase	Y	12.89/14.74	3/3	2/3	38.4	5.85
PDE_04182	Putative acetyl xylan esterase	Y	59.83/51.28	11/6	11/5	24	7.15
PDE_04519	Hypothetical protein	Y	29.63/8.47	3/1	3/1	19.6	4.81
PDE_05305	Hypothetical protein	Y	8.14/10.98	1/2	1/1	59.7	5.17
PDE_05445	Putative cellobiohydrolase	Y	14.57/20.09	5/7	4/1	48.1	4.91
PDE_05537	Hypothetical protein	N	2.34/3.89	1/2	1/1	68.5	5.01
PDE_06023	Putative beta-1,4-mannanase	Y	7.01/41.86	1/7	1/6	47.4	5.33
PDE_06089	Hypothetical protein	Y	30.12/39.76	4/4	4/4	18.1	7.37
PDE_06128	Hypothetical protein	Y	19.21/19.21	4/3	4/3	16.6	5.22
PDE_06138	Ecm33 domain-containing protein	Y	37.66/7.73	17/3	16/3	41.9	5.12
PDE_06252	Hypothetical protein	N	16.42/16.22	8/12	1/1	115.7	7.09
PDE_06439	Putative endo-beta-1,4-glucanase	Y	8.86/37.97	2/6	2/5	25.5	6.52
PDE_06649	Putative feruloyl esterase	Y	11.44/3.23	1/1	1/1	35.7	7.74
PDE_06677	Hypothetical protein	Y	9.26/27.31	2/1	2/1	23.1	5.07
PDE_06697	Hypothetical protein	N	8.65/11.78	4/2	1/1	128.2	7.28
PDE_07033	Hypothetical protein	Y	14.99/4.5	3/1	1/1	50.4	5.34
PDE_07073	Hypothetical protein	N	6.27/2.53	3/2	1/1	118.1	5.86
PDE_07106	Hypothetical protein	Y	44.14/71.03	5/6	4/6	15.1	8.47
PDE_07124	Cellobiohydrolase Cel6A	Y	55.39/56.25	13/22	10/22	48.5	5.67
PDE_07928	Endo-beta-1,4-glucanase Cel45A	Y	9.77/8.65	3/2	3/2	26.9	4.84
PDE_07929	Endo-beta-1,4-glucanase Cel7B	Y	26.37/36.29	8/12	7/10	49.3	5.26
PDE_07938	Putative polygalacturonase	Y	23.72/25.07	6/6	6/5	37.6	5.14
PDE_07945	Cellobiohydrolase CBHI/Cel7A-2	Y	45.05/45.6	22/28	19/25	56.9	5.12
PDE_08075	Hypothetical protein	Y	21.18/5.9	4/1	3/1	27.7	4.02
PDE_08094	Putative endo-beta-1,4-xylanase	Y	23.41/61.95	5/20	5/19	43.5	6.42
PDE_08650	Hypothetical protein	N	6.43/8.55	11/13	1/1	359.1	7.17
PDE_09226	Putative endo-beta-1,4-glucanase	Y	43.1/49.15	13/20	13/20	44	5.21
PDE_09278	Putative acetyl xylan esterase	Y	15.91/31.82	3/7	2/7	41.3	7.84
PDE_09285	Putative alpha-L-rhamnopyranohydrolase	Y	41.08/31.4	12/10	10/9	51.4	4.92
PDE_09289	Hypothetical protein	Y	9.64/9.64	1/2	1/1	18	4.83
PDE_09417	Glucoamylase Amy15A	Y	41.1/51.02	30/25	30/25	67.2	6.01
PDE_09969	Endo-beta-1,4-glucanase Cel5C	Y	21.47/41.5	8/16	7/16	65	5.82

Most of core cellulases were common to both WT and RE-10; thus, we speculated that the difference in secretomes between WT and RE-10 was mainly reflected in proportion and quantity. In the above, one-dimension PAGE and protein concentration analyses revealed the extracellular protein from RE-10 was up-regulated substantially when compared with WT strain (Figures 3F, and 5). Therefore, we then compared the proportion of major cellulolytic enzymes in the secretome between different strains. As expected, the proportion of almost all major cellulases, expected BGL1 was significantly elevated in RE-10 than that of WT, which was consistent with the results from cellulolytic enzyme activities and transcriptional level assays. Specifically, Cel7A-1, Cel7A-2, Cel6A, Cel5B, Cel5C, and Cel7B were up-regulated by 1.39-, 1.26-, 1.68-, 1.3-, 1.99-, and 1.49-fold, respectively, in the secretome of RE-10 compared with those in that of WT (Figure 7B). Furthermore, the ratio of swollenin in RE-10 increased by up to nine times compared with that in WT strain, which was consistent with the qRT-PCR results.

On the contrary, the major extracellular beta-glucosidase BGL1 was undetectable in the secretome of RE-10, and its content was about 2% in the secretome of WT strain. A similar phenomenon was observed in the comparative secretome analysis between industrial strain JU-A10-T and 114-2 [9]. The decline of BGL1 content in hour 96 was consistent with the result from *p*NPGase activity assay on cellulose medium (Additional file 3: Figure S3D), also in agreement with the transcriptional level assay (Figure 6E). However, replacing the cellulose medium with wheat bran medium could alleviate the decrease, suggesting the expression of beta-glucosidases was affected by carbon sources.

## Discussion

In this study, a novel route was developed to rationally redesign the expression regulatory network of cellulolytic fungus *P. oxalicum* for improving cellulase production. Traditionally, random mutagenesis as the main approach is used to construct industrial fungal cellulase producers, which is time-consuming and laborious. Importantly, luck is not always sufficient to obtain the desired mutant by random mutagenesis. Especially, undesired phenotypes accompanied during mutagenesis process were another disadvantage. In *P. oxalicum*, 30 years were devoted to mutagenesis and screening until the high-producer JU-A10-T was obtained. Similarly, the *T. reesei* strain QM6a was discovered during Second World War; however, the cellulase hyper-producer RUT-C30 derived from which was screened until 1979 [36]. By contrast, only about 6 months were spent to construct the triple mutant RE-10, whose cellulolytic ability was parallel to that of industrial strain JU-A10-T (Figures 2, 3, 5, and 6).

We have performed the production of cellulase by RE-10 in 7.5-L bioreactor, and the results exhibited that the strain RE-10 produced higher FPA activity (12 to14 U/mL) than that in flasks, which is expected to be further improved by medium composition and fermentation parameter optimization. Undoubtedly, this study is the most efficient example of the application of genetic modification for achieving cellulase hyper-producer in fungi of the genus of *Penicillium*. To the best of our knowledge, this was also the first report on engineering native filamentous fungus up to the level of an industrial producer in the field of enzyme mixture production.

A growing body of evidence points out that filamentous fungi employ both conserved and unique mechanisms to regulate the production of cellulolytic enzymes. Genomic analysis unveiled that the orthologs of multiple components, including Cre1, ACEI, and LaeA in the cellular mechanism, which control the cellulase production in *T. reesei*, were also present in *P. oxalicum* [6]. On the other hand, species or genus-specific regulators, such as ACEII, Xpp1, ENVOY, and GRD1, were lost in *P. oxalicum*. Lactose, which was always considered as the efficient inducer for cellulase expression in *T. reesei*, also induced the expression of lignocellulolytic genes in *P. oxalicum* at lower concentration [37]. However, sophorose, which is the transglycosylation product of beta-glucosidase, was unable to induce the expression of cellulolytic genes in *P. oxalicum* [20] but intensely stimulated the expression of cellulases in *T. reesei* [38]. Recently, gene perturbation studies in *P. oxalicum* revealed the regulatory role of several proteins in cellulase expression. CreA and its orthologs, as the major repressors for negatively regulating cellulolytic gene expression in *P. oxalicum* [9], and other filamentous fungi, including *T. reesei* and *N. crassa* [16,39], and their deletions are essential to rationally construct cellulase hyper-producer of almost all the cellulolytic fungi. In *P. oxalicum*, our unpublished data found that deletion of the activator ClrB almost block the expression and secretion of cellulases, which is highly similar to the role of its ortholog Clr-2 in *N. crassa* [14], and over-expression of ClrB could lead to a drastic increase in cellulase expression and activities. Resemble the results observed in *T. reesei* [40], the transglycosylation activity of BGL2 was only observed at high substrate concentration in *P. oxalicum* (data not show), and the regulatory role of BGL2 in cellulase production in *P. oxalicum* was basically similar to *N. crassa*, however, clearly different from that of *T. reesei* [18-20]. Our recent studies demonstrated that cellodextrin transporters exhibited functional redundancy in *P. oxalicum*, and deletion of any one of the three (CdtC, CdtD, and CdtG) proteins did not affect cellulase expression [41]; however, lack of either cellodextrin transporter CDT-1 or CDT-2 in *N. crassa* led to notable defects in cellulose utilization [42]. Overall, three regulators in this studies were basically conserved in the three species; the regulatory mechanisms clarified in one fungi can be helpful for genetic engineering of other species for cellulase production improvement.

Just as observed in *A. nidulans* [22], single over-expression of *clrB* in *P. oxalicum* was not sufficient to get rid of the dependence on inducer (data not show). Likewise, a strain carrying *creA*/*cre1* deletion was only partially derepressed in both *P. oxalicum* and *T. reesei* [43], albeit it improved FPA activity by 1.5-fold under induction condition in *P. oxalicum* [9]. Furthermore, deletion *creA* coupling with over-expression activator *clrB* was necessary to get rid of the dependence of inducer, and other double gene mutations including over-expressing *clrB* coupling with deleting *bgl2* and the *bgl2* and *creA* double deletions were able to induce the expression and secretion of cellulase to a higher level (data in our another submitting manuscript); however, their effects were still far less than triple-gene mutants in RE-10, underlining the cumulative effects of the three mutants. Similarly, further lack of Cre-1 in *N. crassa* Δ3βG strain showed higher concentration of secreted active cellulases in Δ3βGΔ*cre* versus Δ3βG in response to induction with cellobiose but not in Avicel [18]. Therefore, we hypothesized that there was a significant synergistic reinforcement effect among the three-step genetic engineering in the induction of cellulolytic gene expression in *P. oxalicum*.

As expected, the mutant RE-10 demonstrated greatly enhanced cellulase activity than WT. However, the extracellular beta-glucosidase activity of RE-10 was less than that of WT, and it was almost undetected at both transcript and protein levels (Figure 6C and Additional file 3: Figure S3D). This phenomenon was only observed under cellulose conditions but not under wheat bran conditions (Figure 3D). The expression of beta-glucosidases is generally believed to be regulated by a unique system that differs from that of other cellulases. BglR, a specific regulator of beta-glucosidases, was recently identified as an activator for efficient beta-glucosidase expression in *T. reesei* [44], but no homolog of *bglR* exists in the genome of *P. oxalicum*. Thus, other novel regulators remain further identification and engineering applications in the near future. Alternatively, the expression of beta-glucosidases may also be improved by placing genes under the promoter from cellulase genes in the future, resulting in co-regulated expression with other cellulases.

Comparative secretome analysis between RE-10 and WT suggested that an up-regulation specifically in lignocellulose-degrading enzymes in the mutant was a consequence of rational and directed engineering. In the secretome of *N. crassa* induced by Avicel, the core cellulase mixture involving cellobiohydrolases, endoglucanase, and β-glucosidase dominated the secretome and comprised 63% to 65% by weight [45], and those homologs in *T. reesei* RUT-C30 account for a larger proportion when induced by lactose [46]. The ratio of the core cellulase mixture was only approximately 24.2% in WT 114-2, and rose to 34.8% in RE-10 when induced by cellulose, which was still lower than that of *N. crassa* or *T. reesei*. Therefore, with the help of recently developed marker gene recycling system (unpublished data), the recombinant RE-10 still holds great potential in the further improvement regarding the content of cellulases in the secretome of *P. oxalicum*.

Strikingly, the protein Cel61A, contained a CBM domain, oxidatively deconstructing cellulose by the aid of redox-active cofactor [47,48], could only be detected in high-producer RE-10 but not in WT. Our results implied that the gene *cel61A* was co-regulated with other core cellulases by the same regulatory network, which accorded with a recent report that a *T. reesei* strain with the gene *cre1* deletion lead to twofold higher over-expression of *cel61A* under cellulose inducing condition [49]. Four GH61 family protein encoding genes were identified in the genome of *P. oxalicum*; two of them were specially detected in RE-10, which were attractive targets in the investigation of alternative lignocellulose-degrading mechanism in filamentous fungi or to supplement the industrial cellulase preparations to improve the efficiency of hydrolysis. In addition, we further confirmed that the protein swollenin was co-regulated with cellulolytic enzymes, as a similar result was previously reported in *T. reesei* [49]. Further, many proteins which are involved in hemicellulose degradation were specially present in the secretome of RE-10, including a putative alpha-L-arabinofuranosidase (PDE_00076), putative beta-1,3-glucanosyltransglycosylase (PDE_03134), putative endo-beta-1,4-xylanase (PDE_05900), and a beta-xylosidase (PDE_02716) (Additional file 5: Table S1). These data conformed to a common sense that expressions of the majority of cellulase and many other plant cell wall-degrading enzymes such as hemicellulase are coordinately regulated in cellulolytic fungi. In addition, a chitin glucanosyltransferase (PDE_05831), a pectin methylesterase (PDE_00923), and a beta-galactosidase were unique to the secretome of RE-10. The latter was previously reported to participate in cellulase induction in *T. reesei* [50]. A comparative analysis of the secreted proteins between WT and cellulase hyper-producers in different fungi will be highly instructive for the identification of conserved and important proteins in lignocellulose degradation. Taken together, all the above knowledge laid a foundation for understanding the unique cellulase system of *P. oxalicum* and provided novel targets for further engineering of *P. oxalicum* and other industrial producers.

## Conclusions

In this study, we genetically modified three key regulators to redesign the regulatory network of the expression of cellulolytic enzymes. The recombinant strain exhibited remarkably strong cellulolytic ability and high cellulase expression and secretion compared with WT. The performance of the recombinant was even comparable with that of industrial strain. Data from comparative proteomics analysis revealed that the lignocellulose-degrading enzymes were elevated specifically in RE-10. Given the conservation of the regulation of cellulolytic enzymes, this novel strategy could be compatible with other cellulolytic fungi.

## Methods

### Strains and culture conditions

*P. oxalicum* 114*-*2 (CGMCC 5302) and all mutants from it maintained on malt extract agar. Cellulose medium comprised 1× Vogel’s salts [51] and 2% microcrystalline cellulose. The wheat bran medium was composed of corn cob residue (2.0000%), Avicel (0.6000%), wheat bran solid (4.6571%), soybean cake powder plate (1.0000%), (NH_4_)_2_SO_4_ (0.2000%), NaNO_3_ (0.2789%), urea (0.1000%), KH_2_PO_4_ (0.3000%), and MgSO_4_ (0.0500%). Cellulase production induced by wheat bran medium and cellulose was performed in a 500 mL flask with 100 mL fluid medium at initial pH 5.5, 30°C, and 200 rpm, using 20 h mycelia pre-grown on 1× Vogel’s medium with glucose (2%, w/v) as a sole carbon source. Cultures were collected by a method of vacuum drum filtration, and 0.5 g vegetative mycelia were added into the above inducing medium. Samples were collected at the time points indicated in the text. Microcrystalline cellulose (CB0279) was purchased from Sangon (Shanghai, China).

### Construction of RE-10 (*gpdA*(p)::*clrB*::*ptra*-Δ*bgl2*::*hph*-Δ*creA*::*bar*) mutant

The cassettes were constructed for gene deletion or over-expression using double-joint PCR [52]. Fungal transformation was conducted by using PEG-mediated protoplast method developed by Li *et al.* [30]. We replaced the *clrB* promoter with *gpdA* (glyceraldehyde-3-phosphate dehydrogenase) promoter from *A. nidulans* [24], using the gene *ptrA* from *A. oryzae* as the selective marker for transformant screening [53]. Using primers PgpdA-F1 and PgpdA-R1, 1314 base pair (bp) of *gpdA* promoter was amplified from plasmid pAN7-1. The 2008 bp *ptra* selectable marker cassette was PCR-amplified with primer pair PtraF1and PtraR1. 3148 bp of *clrB* coding region and terminator was amplified with primer pair clrB-Fa and clrB-Ra, and this fragment overlapped with *gpdA* promoter and *ptra* fragment by 25 bp at its ends, respectively. These 6375 bp PCR products were fused in the order *gpdA*_(p)_-*clrB*_(coding region)_-*ptra* by double-joint PCR with nest primers PgpdA-F2 and PtraR1. One of these transformants, which was verified by PCR with primers PgpdA-F2 and PtraR1, was used for further genetic engineering.

Δ*bgl2*::*hph* knockout cassette with the *hph* cassette was amplified from Δbgl2::hph mutant genome DNA with primer pair Bgl2-F2 + Bgl2-R2 [20]. The Δ*bgl2*::*hph* knockout cassette was used to transform protoplasts of the *gpdA*(p)::*clrB* mutant and obtained Δ*bgl2*-*gpdA*(p)::*clrB* mutants.

Double-joint PCR was performed to construct knockout cassette with the *bar* cassette flanked by 1.5 kb upstream (CreA-F1 and Crebar-R) and 1.5 kb downstream (Crebar-F and CreA-R1) of the *creA* ORF. 1.038 kb of *bar* cassette was amplified from plasmid pUC-bar by using prime pair Bar-F and Bar-R [54]. The final deletion cassette fragment Δ*creA*::*bar* was obtained by using the primer pair (Crenest-F + Crenest-R) with the three fragments above as a template for PCR. The Δ*creA*::*bar* cassettes were transformed into the *P. oxalicum gpdA*(p)::*clrB*-*ptra* and Δ*bgl2* strain protoplasts and obtained the mutant RE-10 *gpdA*(p)::*clrB*::*ptra*-Δ*bgl2*::*hph*-Δ*creA*::*bar*. Transformants were verified by PCR with primer pairs (CreA-F1 + Bar-R, Bar-F + CreA-R1 and Crenest-F + Crenest-R). The primers used in this study were listed in Additional file 6: Table S2.

### Southern blot

The 0.89 kb probe for determining the copy numbers of *clrB* was PCR-amplified from genomic DNA using primers clrb-pF and clrb-pR. The enzymes *Pst*I and *Xba*I were used to digest the genome DNA. Only one 4.3 kb band should be visualized in WT after hybridization, and more than one band should observe in RE-10 (other than the native one). To determine the deletion of *creA*, previous probes and the same restriction enzymes were used [55]. To verify the deletion of *bgl2*, probe was amplified with primers Pbgl2-F and Pbgl2-F, then cleaved the genomes of WT (2.5 kb) and RE-10 (2.9 kb) with enzymes *EcoR*I and *Cla*I, respectively. Labeling of probe, hybridization, and color detection were performed by using DIG High Prime labelling kit (Roche) according to the manufacturer’s methods.

### Phenotype analysis

Microcrystalline cellulose was milled with beads for 6 days at room temperature. For phenotypic analysis, equal volume of conidia (10^8^ per mL) of JU-A10-T, RE-10, and WT were spotted on medium contained 1× Vogel’s salts with 2% glucose or ball-milled cellulose above at 30°C for 4 or 8 days. Canon EOS 600D (Canon, Japan) was used for photographing. We repeated the results and all the above analysis were performed in triplicates.

### Cellulase activity assays

Supernatants were collected by centrifugation for removing mycelia and residual medium. The concentration of total extracellular protein was measured by a Bradford Kit (Sangon, Shang Hai, China), according to the manufacturer’s instructions. FPA and CMCase activities were assayed with filter paper Whatman No. 1 (Shanghai, China) and CMC-NA (Sigma, USA) as the substrates, respectively. The enzyme reactions were performed in 0.2 M acetate buffer (pH 4.8) at 50°C for 60 and 30 min, respectively, using DNS method to quantify the released reducing sugar. Xylanase activity were assayed according to the method described by Sun *et al.* [56]. The *p*NPCase and *p*NPGase activities were measured in the above-used acetate buffer at 30°C for 30 min with *p*NPC and *p*NPG (Sigma, USA) as substrates. One enzyme activity unit was defined as the amount of enzyme required for producing 1 μmol glucose or *p*NP per minute under the assayed conditions. Three biological triplicates were performed in all analyses.

### Biomass and glucose utilization determination

The WT, RE-10, and JU-A10-T strains were pre-grown in 1× Vogel’s medium with glucose (2%, *w*/*v*) at 30, 200 rpm for 20 h. Equal amount of collected mycelia from each strain was transferred to the same media freshly prepared, for another 0, 6, 12, 24, 36, 48, 60, 72, and 84 h, respectively. Buchner funnel was used to separate the mycelia and supernatant. All sampled mycelia trapped into filter paper were drying at 65°C to constant weight. The concentration of glucose was determined by using the biosensor. All analyses were performed in biological triplicates.

### qRT-PCR analysis

Spores of RE-10 and WT strains were washed by the solution containing 0.09 NaCl and 0.1% Twain-20, then inoculated into 1× Vogel’s medium with glucose (2%, w/v) as a sole carbon source at 30°C for 20 h with shaking. Mycelia were collected by filtration and transferred to 1× Vogel’s medium without any carbon source for 2 h, then transferred to inducing medium containing 2% cellulose as a sole carbon source for another 4 or 22 h. Samples of 2 h after starvation, 4 or 22 h after induction were collected. The total RNA extraction and cDNA synthesis using the RNAiso™ reagent (TaKaRa, Japan) and PrimeScript RT Reagent Kit (TaKaRa, Japan) were performed according to the manufacturer’s instructions. qRT-PCR was performed on the LightCycler instrument (Roche, Germany) with software Version 4.0 (Roche, Germany) as previously described [41]. The primers used are shown in Additional file 6: Table S2. At least two biological triplicates were performed, and qRT-PCR of each gene was performed in three triplicates. The expression of actin was chosen as the reference gene for data normalization. The relative expression level was defined as follows: Rel. expression level (gene X) = copy number of gene X/copy number of gene action.

### Protein gel electrophoresis

Unconcentrated supernatants were added to loading buffer, boiled for 5 min for degeneration, and loaded onto a 12% Tris-HCl polyacrylamide gel. Coomassie blue stain reagent was used for staining.

### Zymography analysis

The 4-methylumbeliferyl-β-d-cellobioside [30] (Sigma) was used as the substrate to detect the cellobiohydrolase activities. Culture supernatant (equal protein) was separated on the polyacrylamide gels (SDS-PAGE) on ice at 138 V for 1 h. After electrophoresis, the gel was washed in 0.2 M acetate buffer (pH 4.8) with 5% Triton X-100 at least for three times to remove the SDS to renature the protein, then washed twice in acetate buffer at room temperature. The gel was directly soaked into acetate buffer containing 0.1% MUC and shaked at 50°C for 1 h, then visualized under UV illumination. And the software Image J (BioRad, USA) was used to obtain the pictures.

### Proteomics analysis

Fresh spores were inoculated into 100 mL of 1× Vogel’s salts supplemented with 2% glucose in a 500 mL Erlenmeyer flask for 24 h and transferred to 2% cellulose medium for 96 hour. The supernatant was collected by filtration through a 0.22 μm PES membrane, concentrated, and desalted by a centrifugal concentrator with a molecular cut-off of 10 kDa (Pall Corporation). The samples were precipitated by acetone and trichloroacetic acid (20:1). Dry protein powders were dissolved in denaturation buffer (0.5 M Tris-HCl, 2.75 mM EDTA, 6 M guanadine-HCl) and reduced by 1 M dithiothreitol at 37°C for 1 h. Alkylation was performed using iodoacetamide for 2 h in the dark. The alkylated samples were desalted and collected by a Microcon YM-10 Centrifugal Filter (Millipore Corporation, USA) according to the manufacturer’s instruction. The collected protein samples were digested by trypsin (Sigma, USA) at 37°C overnight. Digested peptides were desalted and collected by a ZipTip C18 column (Millipore Corporation, USA). The collected secretome samples were separated on a C18 reversed phase column (15 cm long, 75 μm inner diameter, packed in-house with ReproSil-Pur C18-AQ 3 μm resin, provided by Dr. Maisch) directly mounted on the electrospray ion source of a mass spectrometer. The peptides were subjected to nanoelectrospray ionization, followed by tandem mass spectrometry (MS/MS) in an LTQ Orbitrap Velos Pro (Thermo Scientific™, USA) coupled inline to HPLC. Intact peptides were detected in the Orbitrap at a resolution of 60,000. Peptides were selected for MS/MS using collision-induced dissociation operating mode with a normalized collision energy setting of 35%. Ion fragments were detected in the LTQ. A data-dependent procedure that alternated between one MS scan followed by ten MS/MS scans was applied for the ten most abundant precursor ions above a threshold ion count of 5,000 in the MS survey scan with the following dynamic exclusion settings: repeat counts, 2; repeat duration, 30 s; and exclusion duration, 120 s. The applied electrospray voltage was 2.2 kV. For MS scans, the m/z scan range was 350 Da to 1,800 Da. MS data processing was performed using Mass-Lynx software (version 4.1, Waters, USA). Proteins with high confidence (*P* <0.01) or at least two peptides detected were collected for further analysis. Data resulting from LC-MS/MS analysis of trypsin-digested proteins were searched against the *P. oxalicum* protein database as previously described [6]. Functional matching of identified proteins was conducted using SEQUEST. SignalP 4.1 (http://www.cbs.dtu.dk/services/SignalP/#citations), SecretomeP 2.0 (http://www.cbs.dtu.dk/services/SecretomeP/), and WoLF PSORT (http://www.genscript.com/psort/wolf_psort.html) with default cut-off values were used to predict the secreted proteins. The ratio of individual protein was calculated by the number of peptides divided by the total peptides of all proteins in the secretome.

### Statistical analysis

A *t*-Student one-tail test for paired samples was performed with the software Microsoft Office 2013 Excel (Microsoft, USA). The mean values, standard deviations, and *P* values were calculated in all quantitative analysis.

### Accession numbers

The GenBank accession numbers for the three proteins manipulated in this study are as follows: ClrB, EPS31045; BGL2, EPS25645; CreA, EPS28222.

## Additional files

Additional file 1: Figure S1. Southern blot analysis of all mutants of RE-10. (A) *clrB* over-expression, (B) *bgl2* deletion, (C) *creA* deletion. Additional file 2: Figure S2. qRT-PCR analysis of the transcripts of manipulated regulator genes (A) *clrB*, (B) *bgl2*, (C) *creA*. Additional file 3: Figure S3. Comparative cellulolytic activity assay in cellulose medium. The FPA (A), *p*NPCase (B), CMCase (C), *p*NPGase (D), xylanase activities (E), and protein (F) of WT and RE-10 on cellulose medium were determined. Additional file 4: Figure S4. SDS-PAGE analysis of the secreted protein. Additional file 5: Table S1. Proteins identified in the secretome of WT and RE-10. Additional file 6: Table S2. Primers used in this study.

## Abbreviations

Bgl2: beta-glucosidase 2
CBM: cellulose binding domain
CDT/Cdt: cellodextrin transporter
ClrB: cellulase regulator B
CMC: carboxymethylcellulose
CreA: carbon catabolite repressor A
FPA: filter paper activity
*p*NPC: 4-Nitrophenyl-β-D-cellobioside
*p*NPG: 4-Nitrophenyl-β-D-glucopyranoside
WT: wild type
