# Supplementary figures and images for: Redesigning the regulatory pathway to enhance cellulase production in Penicillium oxalicum
Source: Biotechnol Biofuels. 2015 Apr 23;8:71. doi: 10.1186/s13068-015-0253-8 (PMC4422585; doi:10.1186/s13068-015-0253-8)

**Fig. 1**


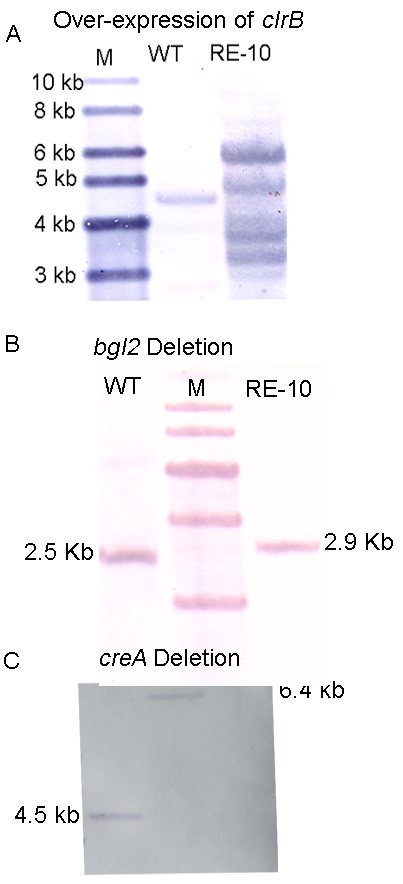

Supplement: Additional file 1: Figure S1. — Southern blot analysis of all mutants of RE-10. (A) clrB over-expression, (B) bgl2 deletion, (C) creA deletion. [file 13068_2015_253_MOESM1_ESM.docx]

**Fig. 2**

**A**

**
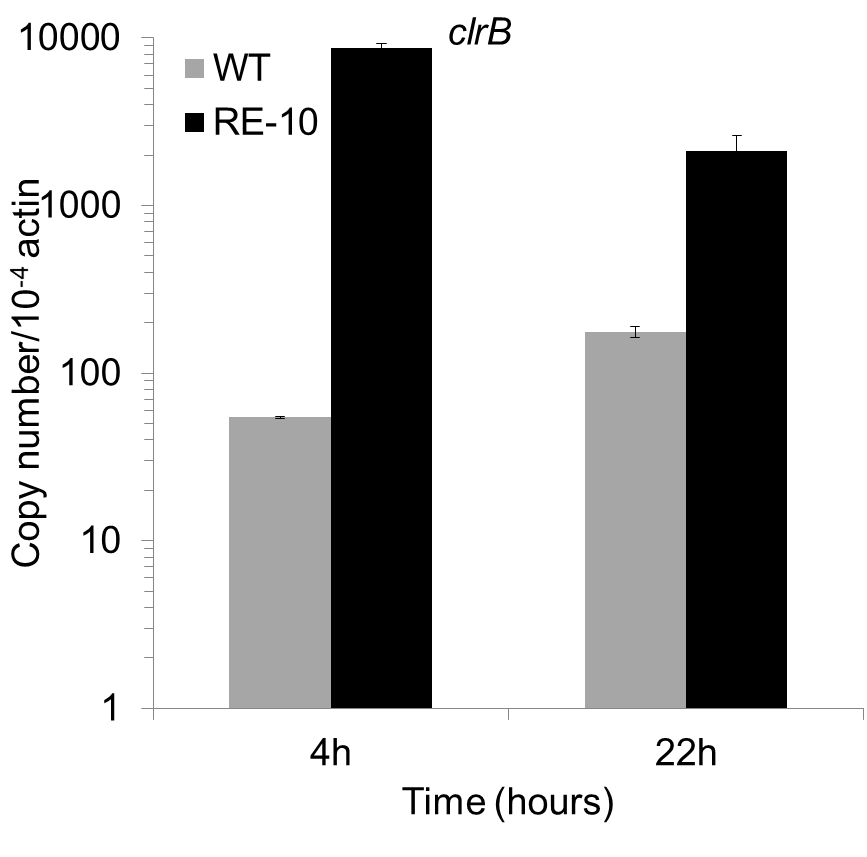
**

**B**

**
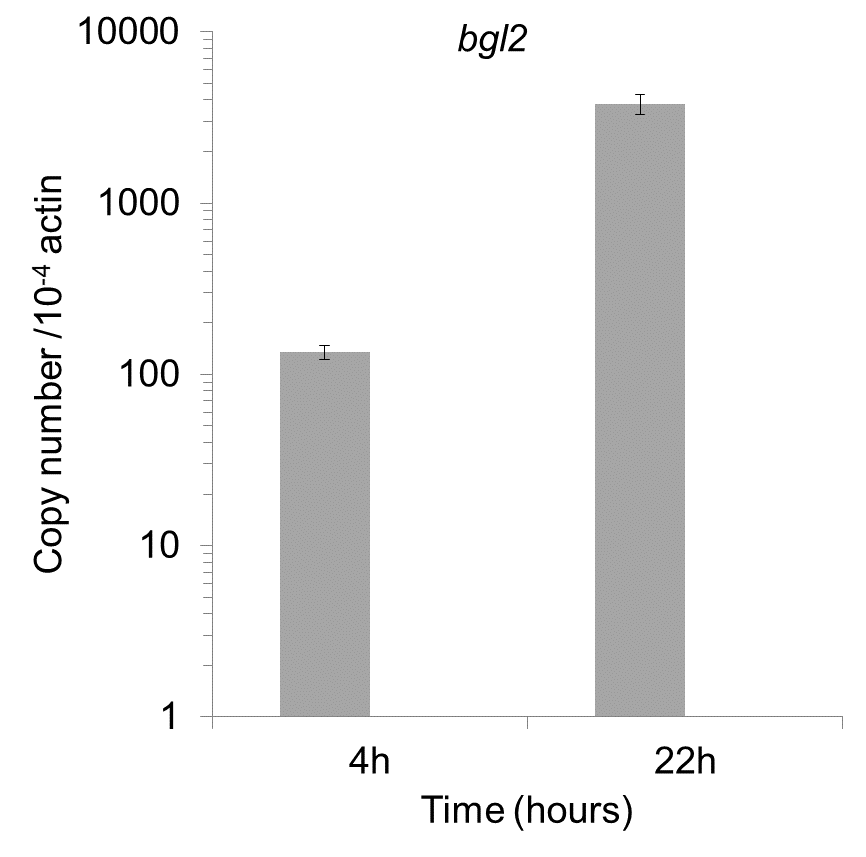
**

**C**

**
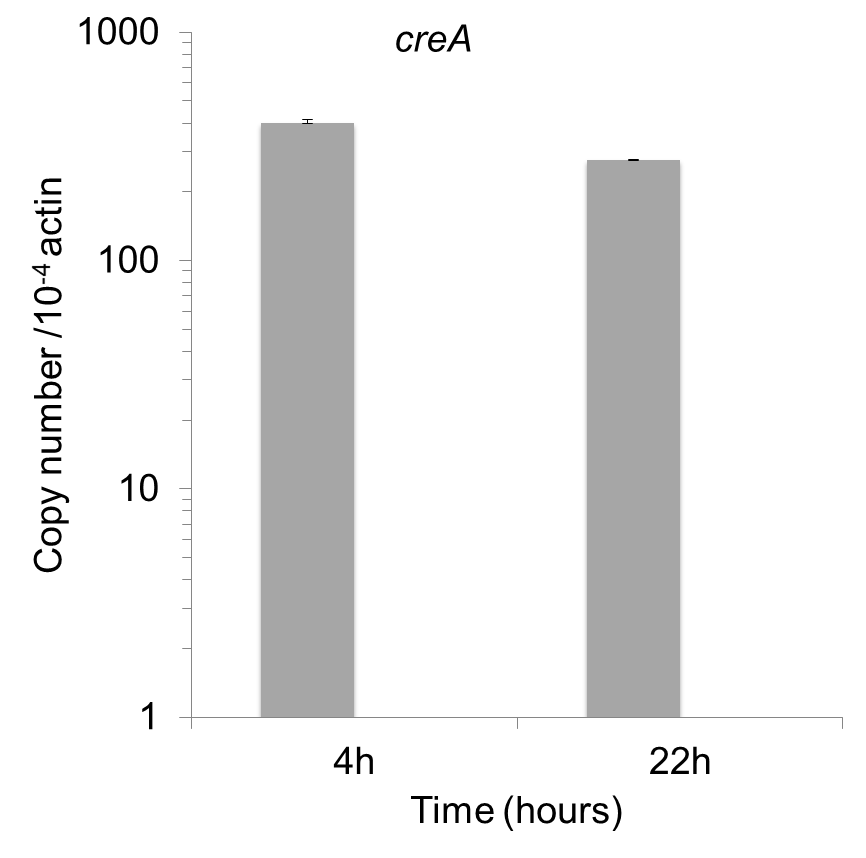
**

Supplement: Additional file 2: Figure S2. — qRT-PCR analysis of the transcripts of manipulated regulator genes (A) clrB, (B) bgl2, (C) creA. [file 13068_2015_253_MOESM2_ESM.docx]

**Fig. 3**

**A B**

**
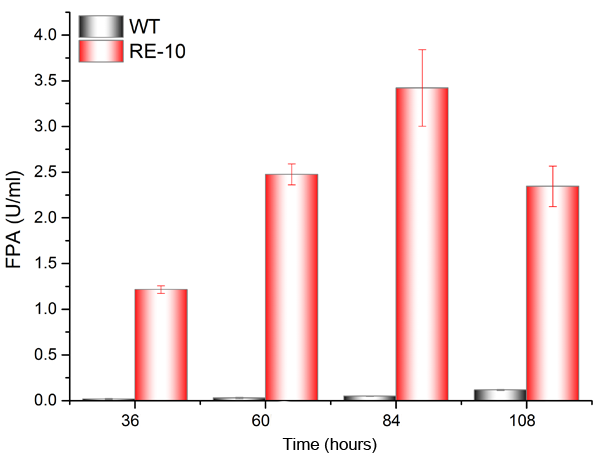

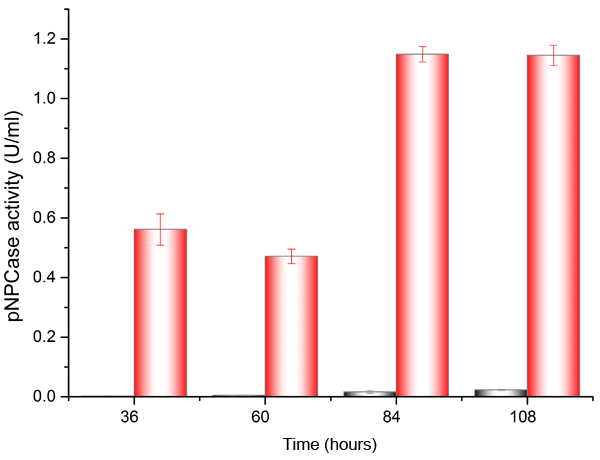
**

**C D**

**
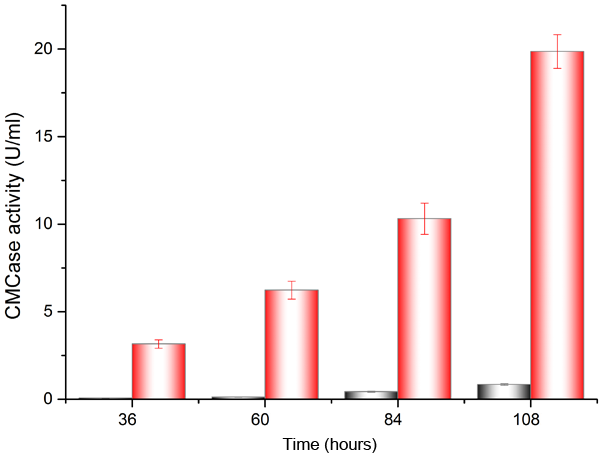

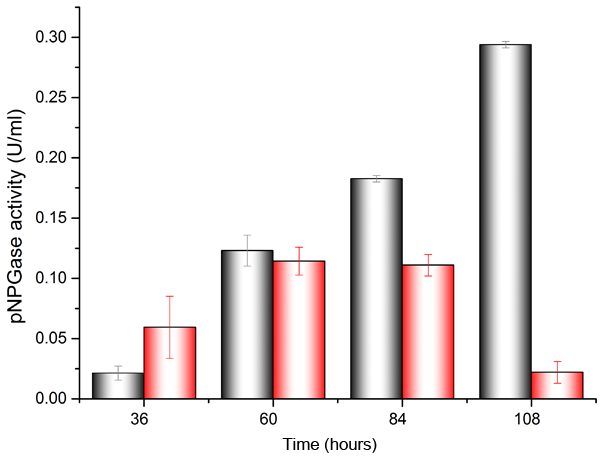
**

**E F**

**
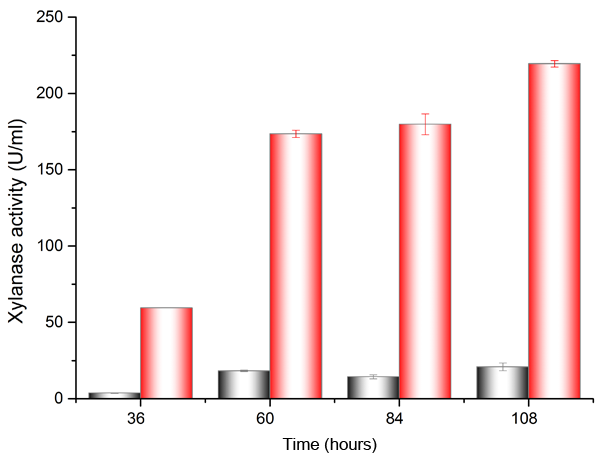

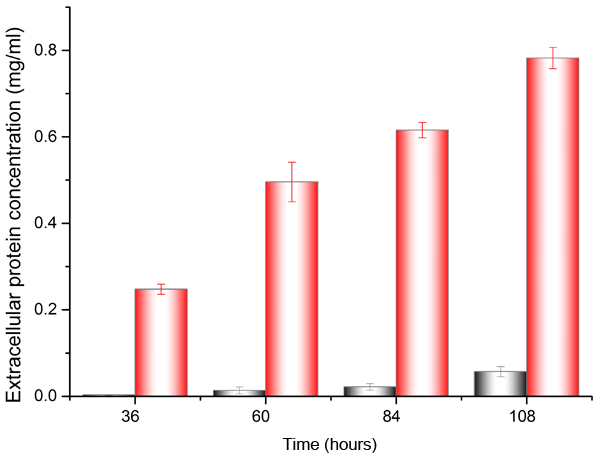
**

Supplement: Additional file 3: Figure S3. — Comparative cellulolytic activity assay in cellulose medium. The FPA (A), pNPCase (B), CMCase (C), pNPGase (D), xylanase activities (E), and protein (F) of WT and RE-10 on cellulose medium were determined. [file 13068_2015_253_MOESM3_ESM.docx]

**Fig. 4**


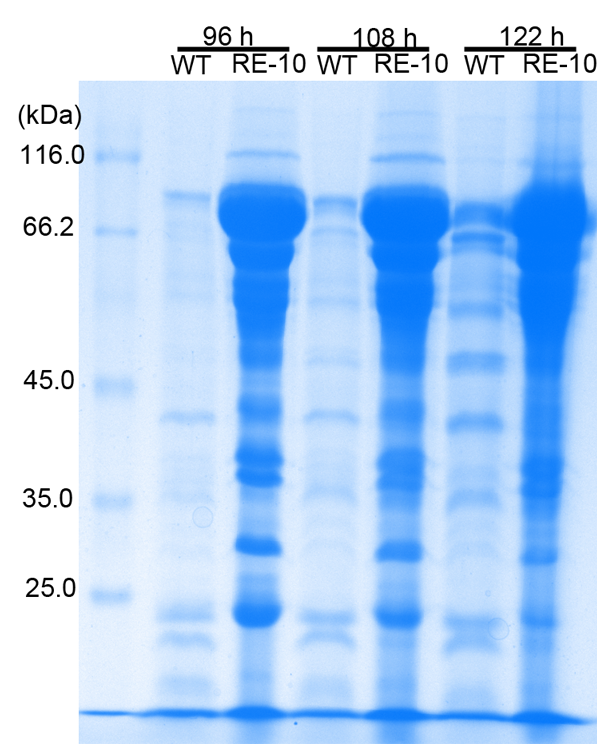

Supplement: Additional file 4: Figure S4. — SDS-PAGE analysis of the secreted protein. [file 13068_2015_253_MOESM4_ESM.docx]
