# Supplementary material for: Redesigning the regulatory pathway to enhance cellulase production in Penicillium oxalicum
Source: Biotechnol Biofuels. 2015 Apr 23;8:71. doi: 10.1186/s13068-015-0253-8 (PMC4422585; doi:10.1186/s13068-015-0253-8)
Supplement: Additional file 6: Table S2. — Primers used in this study. [file 13068_2015_253_MOESM6_ESM.docx]

Additional file 6: Table S2**.** Primers used in this study.

| Primer name | Sequence (5′-3′) |
| --- | --- |
| Primers for *clrB* overexpression | |
| PgpdA-F1 | AGTCAGACGGCGTAACCAAA |
| PgpdA-R1 | GGTGATGTCTGCTCAAGCGG |
| clrB-Fa | AACAGCTACCCCGCTTGAGCAGACATCACCATGTTCCACACCTTTGAAGG |
| clrB-Ra | CCAATGGGATCCCGTAATCAATTGCCCGTAGCACCAGCGAAACATAC |
| PtraF1 | gggcaattgattacgggatc |
| PtraR1 | gcggctcatcgtcaccccat |
| PgpdA-F2 | GTAAGGATTTCGGCACGG |
| Primers for deletion of *creA* | |
| Cre1-F1 | CATTCCAGAGATGAACGACC |
| Crebar-R | GTAAGCGAATTAGCAAGCGTCGATGTGGGAACACCGGA |
| Crebar-F | GAGGGCAGCAAGCCAGTGCactctcacgactcattgctcg |
| Cre1-R1 | CCAGAGGATGGAACAAACAC |
| Crenest-F | TCAGTTTGATGCCCCTATCT |
| Crenest-R | TCCCTCGCCACTTCGGAAAC |
| Bar-F | GACGCTTGCTAATTCGCTTAC |
| Bar-R | GAGGGCAGCAAGCCAGTGC |
| Primers for q-PCR | |
| Cel7A-2F | CCACCACCACTACCAGCAAGG |
| Cel7A-2R | GTAGCCAACACCACCGCACT |
| Cel5B-F | ACCGCTGCTCAGACCACGAC |
| Cel5B-R | TGGGTCCCGAGTAGCCAACG |
| Bgl1-F | CACCAACACCGGCTCAGTTA |
| Bgl1-R | GGACATCCCAGTTGGACAGAT |
| Swo-F | AACGCTATGGTGCTTGGGTG |
| Swo-R | GGCTGGTGCAGTGAAGGAAG |
| Cel61-F | GCCTCAGGGTACTTTGGGTG |
| Cel61-R | TGGACCGGGAATCACGTAGC |
| ClrB-F | CCATCGAATCTTGCCAAGCAC |
| ClrB-R | GCTTGCTGGCTTCGTAAATGC |
| Cre-F | TGTCCCGCAGGTCCCTAAAGT |
| Cre-R | GCCCGCCACGGAATTATTTGT |
| Bgl2-F | GGCTGATGCGTACACGTTTGA |
| Bgl2-R | CGACATAAGTCACGCCGAAGC |
| Actin-F | CGACATAAGTCACGCCGAAGC |
| Actin-R | AGAAGCACTTGCGGTGAACGA |
| Probe primers | |
| clrb-pF | ATGTTCCACACCTTTGAAGG |
| clrb-pR | ATTTCGGTCGGAGGGATGA |
| Pbgl2-F | AGATGATGAATCCGAAGATGAAGAC |
| Pbgl2-R | CAGCACAACGGAGACCTACA |

^a^ The overlapped sequence is underlined.
